# Methicillin-Resistant and -Susceptible *Staphylococcus aureus* Sequence Type 398 in Pigs and Humans

**DOI:** 10.3201/eid1403.0760

**Published:** 2008-03

**Authors:** Alex van Belkum, Damian C. Melles, Justine K. Peeters, Willem B. van Leeuwen, Engeline van Duijkeren, Xander W. Huijsdens, Emile Spalburg, Albert J. de Neeling, Henri A. Verbrugh, on behalf of the Dutch Working Party on Surveillance Research of MRSA (SOM)

**Affiliations:** * University Medical Center Rotterdam, Rotterdam, the Netherlands; † University of Utrecht, Utrecht, the Netherlands; ‡ National Institute for Public Health and the Environment (RIVM), Bilthoven, the Netherlands; 1 Members of SOM: A. van Belkum, M. Bonten, M. van den Broek, J. Degener, E. van Duijkeren, A. van de Giessen, X.W. Huijsdens, J.A.J.W. Kluytmans, B. ter Kuile, I. van Loo, D. Mevius, A.J. de Neeling, R. van Oosterom, E. Stobberingh, E.W. Tiemersma, H.A. Verbrugh, A. Voss, J.A. Wagenaar, and P. van der Wolf

**Keywords:** Staphylococcus aureus, MRSA, sequence type 398, virulence, nasal carriage, dispatch

## Abstract

Methicillin-resistant *Staphylococcus aureus* sequence type 398 (ST398 MRSA) was identified in Dutch pigs and pig farmers. ST398 methicillin-susceptible *S. aureus* circulates among humans at low frequency (0.2%) but was isolated in 3 human cases of bacteremia (2.1%; p = 0.026). Although its natural host is probably porcine, ST398 MRSA likely causes infections in humans.

Nasal *Staphylococcus aureus* carriage has increased in pig farmers, and specific lineages of *S. aureus* are shared by farmers and their animals (*1*,*2*). In addition, rates of nasal carriage of methicillin-resistant *S. aureus* (MRSA) by veterinary personnel working with pigs is high (*3*–*5*). The pig-related MRSA appears to be clonal and was identified by multilocus sequence typing (MLST) as sequence type 398 (*2*,*6*,*7*). Such resistant bacterial strains can spread from animals to the environment, which may facilitate the colonization of persons who are not involved in animal husbandry (*8*). The porcine MRSA strain has been isolated from humans with invasive and superficial infections, and familial outbreaks of colonization and cross-colonization have been documented (*2*,*6*,*7*).

We sought to determine whether the clinical effect of the porcine ST398 MRSA strain can be substantiated by the existence of genetically homologous, methicillin-susceptible *S. aureus* (MSSA) strains among healthy or infected persons. The international MLST database (www.mlst.net) (*9*) listed only 1 ST398 MSSA nasal carriage isolate from a patient in Cape Verde. In addition, 1 ST398 MRSA strain was isolated from a woman living in Groningen, the Netherlands, without further clinical and epidemiologic data available. ST398 MSSA nasal carriage isolates were also identified in several pig farmers in a study by Armand-Lefevre et al. (*1*). We describe the population genetic analysis of Dutch community-based and nosocomial MSSA isolates in comparison with pig- and pig farmer–derived ST398 MRSA isolates, performed by *spa*-sequencing and amplified fragment length polymorphism (AFLP) analysis (*10*,*11*).

## The Study

Most of the ST398 MRSA strains studied were collected at the Dutch Institute for Public Health and the Environment (RIVM, Bilthoven, the Netherlands). A total of 20 strains were isolated from the nares of pigs in several slaughterhouses (RIVM 21–40) (*12*), whereas 18 additional strains were detected during in-hospital screenings for MRSA carriage among Dutch farmers from independent farms (RIVM 1–8, 10–12 and 14–20). In addition, 8 clinical and carriage isolates were obtained from the Veterinary Medical Diagnostic Centre in Utrecht (Table).

**Table:** Survey of demographic and clinical data of the ST398 pig-associated MRSA (n = 46) and ST398 MSSA (n = 6) strains, the Netherlands*

Isolate no.	Source	Infection	Site of sampling	City	*mec*A	PVL	*spa* type	SCC*mec*
RIVM-1	Human	No	Nose	Putten	Pos	Neg	t011	IVa
RIVM-2	Human	Yes	Urine	Rosmalen	Pos	Neg	t108	V
RIVM-3	Human	No	Nose	Gendringen	Pos	Neg	t108	V
RIVM-4	Human	Yes	Wound/abscess	Helden	Pos	Neg	t108	V
RIVM-5	Human	No	Sputum	Amsterdam	Pos	Neg	t011	V
RIVM-6	Human	Yes	Urine	Zeeland	Pos	Neg	t108	V
RIVM-7	Human	No	Throat/nose	Bennekom	Pos	Neg	t898	III
RIVM-8	Human	No	Sputum	Kootwijkerbroek	Pos	Neg	t011	V
RIVM-10	Human	Yes	Wound/abscess	Galder	Pos	Neg	t567	III
RIVM-11	Human	Yes	Wound/abscess	Olburgen	Pos	Neg	t108	V
RIVM-12	Human	No	Sputum	Sas van Gent	Pos	Neg	t011	IVa
RIVM-14	Human	Yes	Nose	Siebengewald	Pos	Neg	t108	V
RIVM-15	Human	No	Sputum	Grijpskerke	Pos	Neg	t034	V
RIVM-16	Human	No	Sputum	Harskamp	Pos	Neg	t108	V
RIVM-17	Human	Yes	Wound/abscess	Haarlem	Pos	Pos	t034	III
RIVM-18	Human	Yes	Sputum	Heeswijk dinther	Pos	Neg	t108	III
RIVM-19	Human	Yes	Perineum	Veenoord	Pos	Neg	t571	III
RIVM-20	Human	Yes	Wound/abscess	Roggel	Pos	Neg	t108	V
RIVM-21	Pig	No	Nose	Venray	Pos	Neg	t108	V
RIVM-22	Pig	No	Nose	Heeze-leende	Pos	Neg	t108	V
RIVM-23	Pig	No	Nose	Barneveld	Pos	Neg	t011	IVa
RIVM-24	Pig	No	Nose	Dalfsen	Pos	Neg	t108	V
RIVM-25	Pig	No	Nose	Gemert-bakel	Pos	Neg	t108	V
RIVM-26	Pig	No	Nose	Venray	Pos	Neg	t108	V
RIVM-27	Pig	No	Nose	Venray	Pos	Neg	t011	V
RIVM-28	Pig	No	Nose	Alphen-chaam	Pos	Neg	t011	IVa
RIVM-29	Pig	No	Nose	Hoogeveen	Pos	Neg	t1254	IVa
RIVM-30	Pig	No	Nose	Skarsterlân	Pos	Neg	t1254	IVa
RIVM-31	Pig	No	Nose	Someren	Pos	Neg	t011	V
RIVM-32	Pig	No	Nose	Someren	Pos	Neg	t1255	V
RIVM-33	Pig	No	Nose	Zundert	Pos	Neg	t108	V
RIVM-34	Pig	No	Nose	Baarle-nassau	Pos	Neg	t108	V
RIVM-35	Pig	No	Nose	Hofvantwente	Pos	Neg	t011	IVa
RIVM-36	Pig	No	Nose	Zwolle	Pos	Neg	t011	IVa
RIVM-37	Pig	No	Nose	Alphen-chaam	Pos	Neg	t011	V
RIVM-38	Pig	No	Nose	Ijselstein	Pos	Neg	t011	IVa
RIVM-39	Pig	No	Nose	Ijselstein	Pos	Neg	t011	IVa
RIVM-40	Pig	No	Nose	Dalfsen	Pos	Neg	t567	III
6302/3	Pig	Yes	Skin	Utrecht	Pos	Neg	t011	IV
6303/6	Pig	Yes	Nose	Utrecht	Pos	Neg	t011	IV
6303/7	Pig	Yes	Nose	Utrecht	Pos	Neg	t011	IV
6303/9	Pig	No	Nose	Utrecht	Pos	Neg	t011	IV
6303/11	Pig	No	Nose	Utrecht	Pos	Neg	t011	IV
6303/1 MRSA1	Human	No	Nose	Utrecht	Pos	Neg	t011	IV
6303/1 MRSA2	Human	No	Nose	Utrecht	Pos	Neg	t011	IV
V0606303/8	Pig	Yes	Nose	Utrecht	Pos	Neg	t011	IV
T0976	Human	No	Nose	Rotterdam	Neg	Neg	t571	–
TE5029	Human	No	Nose	Rotterdam	Neg	Neg	t571	–
TB27855	Human	Yes	Blood	Rotterdam	Neg	Neg	t571	–
TB28395	Human	Yes	Blood	Rotterdam	Neg	Neg	t571	–
TB29854	Human	Yes	Blood	Rotterdam	Neg	Neg	t571	–
TDV0002580	Gorilla	Yes	Ear	Velp	Neg	Neg	t034	–

*MRSA; methicillin-resistant *Staphylococcus aureus*; MSSA, methicillin-susceptible *S. aureus*; ST398, sequence type 398; PVL, Panton-Valentine leukocidin; SCC*mec*, staphylococcal chromosome cassette; pos, positive; neg, negative. All isolates were nontypeable by pulsed-field gel electrophoresis and confirmed as ST398 by multilocus sequence typing.

Amplified fragment length polymorphism (AFLP) analysis was performed as described previously (*10*). A total of 147 marker fragments per strain were scored, and a binary table with marker absence [0] or presence [1] was constructed. A total of 30 fragments with differential occurrence, when genetically heterogeneous MSSA and ST398 MRSA fingerprints were compared, were reamplified and sequenced (Applied Biosystems, Foster City, CA, USA). Fragments were sequenced for 3 independent strains, and the consensus was analyzed by using BLAST (www.ncbi.nlm.nih.gov/blast). Typing of the staphylococcal chromosome cassette (SCC*mec*) and the presence of the Panton-Valentine leukocidin (PVL) genes was performed by PCR.

We embedded the genetic fingerprints of the 46 pig-related MRSA isolates in the population structure of *S. aureus* as obtained before (*10*,*11*). These studies include high throughput AFLP fingerprints of 829 nonclinical *S. aureus* human carriage isolates and 146 and 77 (including 2 MRSA isolates) clinical isolates of human and animal origin, respectively. All carriage strains were isolated from volunteers living in the Rotterdam region, where pig farms are absent.

Sequencing of the repetitive region of the protein A gene *spa* was performed for all ST398 MRSA isolates (*13*). Data were analyzed by using the Ridom Staphtype software version 1.4 (www.ridom.de/staphtype).

Analysis of the AFLP data was performed as described previously (*10*). Both hierarchical cluster analysis and principle component analysis were performed with Spotfire DecisionSite 7.2 software (www.spotfire.com). We used the Fisher exact test to compare the distribution of strain categories in different phylogenetic lineages. A 2-sided p value <0.05 was considered significant.

**Figure 1:**
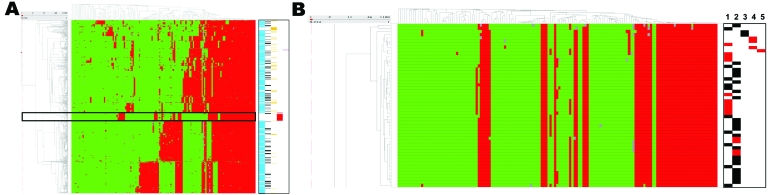
A) Meta-analysis of the amplified fragment length polymorphism data obtained for the pig-associated methicillin-resistant *Staphylococcus aureus* sequence type 398 (ST398 MRSA) and its closely related methicillin-susceptible *S. aureus* (MSSA) strains, carriage MSSA isolates from healthy children and elderly persons, invasive MSSA from hospitalized children and elderly persons, and invasive animal *S. aureus* isolates (including 2 MRSA isolates) (*10**,**11*). Green and red represent 161,700 binary outcomes generated by high throughput restriction fragment length polymorphism with 147 marker fragments. Marker absence corresponds with green, marker presence corresponds with red, and gray represents ambiguous positions (i.e., weak bands), scored as marker absence in the mathematical analyses. ST398 MRSA strains are boxed. The dendrogram on the left shows the phylogenetic strain clustering; the dendrogram on the x-axis shows marker clustering. Marker groups are cluster specific. Markers on the right are defined as follows: blue, carriage isolates (n = 829); black, bacteremia isolates (n = 146); yellow, animal isolates (n = 77); red, ST398 MRSA isolates (n = 46); pink, reference strains (Mu50/N315). B) Detail highlighting the ST398 isolates. Markers and lanes on the right are defined as follows: black, carriage isolate; red, clinical isolate; 1, ST398 MRSA isolated from humans; 2, ST398 MRSA isolated from pigs; 3, ST398 MSSA human carriage isolates; 4, ST398 MSSA human bacteremia isolates; 5, ST398 MSSA animal clinical isolate.

The AFLP analysis of the ST398 MRSA strains derived from human and animal sources (n = 46) indicated that these strains are highly clonal. When the AFLP patterns for the ST398 strains were included in the overall population analysis for Dutch MSSA strains from carriage and infection, the distinct cluster was still observed (Figure 1). Few Dutch MSSA strains from the Rotterdam region coclustered with the ST398 pig-related MRSA isolates (Figure 1, **panel B**). In total, 6 (0.6%) MSSA isolates coclustered with the ST398 MRSA isolates, of which 2 were nasal carriage isolates from healthy persons (Table). Of the 6 strains, 3 were blood culture isolates taken from 3 elderly patients. All 3 patients had nosocomial bacteremia: 1 after inflammatory aneurism of the aorta, 1 during Fournier gangrene, and the last 1 after primary ventricular fibrillation. Epidemiologic data exclude a cluster of nosocomial infections; patients were not in direct contact (data not shown).

**Figure 2:**
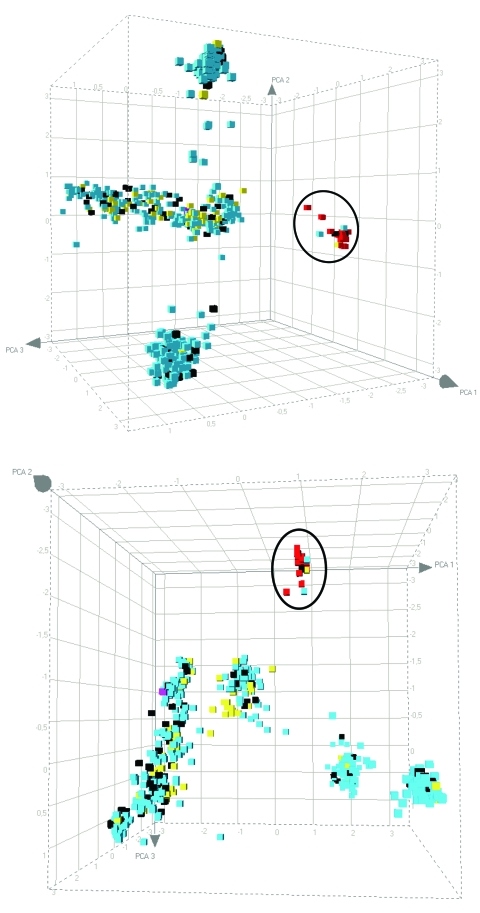
Principal component analysis analysis of the amplified fragment length polymorphism data obtained for the pig-associated methicillin-resistant *Staphylococcus aureus* sequence type 398 (ST398 MRSA) and its closely related methicillin-susceptible *S. aureus* (MSSA) strains, carriage MSSA isolates from healthy children and elderly persons, invasive MSSA from hospitalized children and elderly persons, and invasive animal *S. aureus* isolates (including 2 MRSA isolates). The cubes, plotted in 3-dimensional space, represent all of the strains displayed in Figure 1, panel A. Each axis represents the score calculated for that strain on each principal component. The distribution is shown from 2 different angles. ST398 strains are circled. Blue, carriage isolates (n = 829); black, bacteremia isolates (n = 146); yellow, animal isolates (n = 77); red, ST398 MRSA isolates (n = 46); pink, reference strains (Mu50/N315).

After principle component analysis , the ST398 MRSA strains still clustered as a separate group (Figure 2). The AFLP analysis did not distinguish strains from pigs or pig farmers, and only a limited number of polymorphic AFLP fragments were seen. AFLP markers that were positive for the ST398 MRSAs and absent from the other strains, or vice versa, were sequenced. Of 30 fragments analyzed, 9 were ≈100% specific for the pig-associated strains. Another 3 fragments were present in a subset of the pig-associated strains only. Of these 12 fragments, 4 were not homologous with current entries in the GenBank database, including the 10 *S. aureus* full-genome sequences. Of the 12 pig-specific markers, 8 were homologous with known sequences, which suggests that these markers become pig-specific by point mutations in the AFLP primer annealing site(s) rather than by genomic rearrangement. Several of the sequences encode factors were associated with membranes or transport.

The preponderance of types t011 and t108 was confirmed by *spa* sequencing (*12*). These made up >75% of all cases. However, the other types all belonged to the same family of *spa* types, which suggests recent drift in the sequence motifs. The t011 types are primarily associated with SCC*mec* IV and IVa, whereas the t108 type is nearly fully associated with SCC*mec* V. This finding suggests that ST398 MRSA has arisen independently on at least 2 occasions. Finally, SCC*mec* III is found in association with t108, t898, t567, t034, and t571. This finding suggests promiscuous dissemination of this cassette among the ST398 MRSA. Strain RIVM-17 harbored the PVL genes. Apparently, the bacteriophage carrying these genes found its way into the porcine ST398 MRSA lineage.

## Conclusions

The massive colonization of Dutch pigs with a single sequence type of MRSA was unexpected (*12*). Molecular strain typing was initially compromised because PFGE failed (*14*). *Spa* gene sequencing (*13*) showed heterogeneity in the ST398 MRSA lineage with types t011 and t108, which are closely related, covering >75% of all isolates. Hence, 1 or 2 new MRSA lineages had been discovered. We found a degree of genetic association between *spa* types and the presence of certain SCC*mec* cassettes, which suggests bacterial evolution and horizontal DNA exchange in the zoonotic reservoir.

We found that ST398 is rare among Dutch MSSA strains colonizing healthy persons (2 [0.2%] of 829 strains). However, a relatively high number of MSSA isolates homologous to the ST398 MRSA were derived from bacteremic patients (3 [2.1%] of 146; p = 0.026). These 3 bacteremia isolates were not related epidemiologically; they were isolated from different patients in different medical departments over an extended period. This finding suggests that these MSSA strains are quite virulent. The strict segregation of ST398 strains (Figure 1, **panel A**; Figure 2) corroborates that the strains belong to a separate biotype associated with pigs (*15*).

Our findings pose a warning to public health surveillance: if the ST398 MSSA virulence toward humans would be maintained within the ST398 MRSA lineage from pigs, care should be taken not to introduce this strain into humans. We consider it to be likely that ST398 MRSA from pigs is capable of causing serious infection in humans even though its primary host seems to be pigs.
